# Evaluation of superficial tumor treatment using radixact with the kilovoltage computed tomography system: dosimetric and clinical feasibility of super stuff bolus

**DOI:** 10.3389/fonc.2025.1707822

**Published:** 2025-11-27

**Authors:** Ryuichi Yada, Yuta Omi, Tatsuya Hasegawa, Katsumasa Nakamura

**Affiliations:** 1 Department of Regional Medical Management Studies, Hamamatsu University School of Medicine, Hamamatsu, Shizuoka, Japan; 2 Department of Radiation Oncology, Anjo Kosei Hospital, Anjo, Aichi, Japan; 3 Department of Radiation Oncology, Hamamatsu University School of Medicine, Hamamatsu, Shizuoka, Japan

**Keywords:** super stuff bolus, surface dose measurement, long-term stability, radixact, kilovoltage computed tomography system, air gap, clinical practice, metastatic inguinal lymphnode lesions of Ewing’s sarcoma

## Abstract

**Background:**

Flat commercial boluses for superficial tumor radiation therapy often fail to conform to irregular body surfaces, resulting in air gaps that reduce dose coverage and uniformity. Although three-dimensional printed custom boluses have been developed to address this issue, the plastic material rigidity and time-consuming fabrication process limit their application. This study aimed to evaluate a flexible and easily moldable Super Stuff bolus as a practical alternative.

**Methods:**

We conducted a three-part study using Radixact with the kilovoltage computed tomography (kVCT) system. First, surface dose measurements were performed using radiochromic film on a solid water phantom. Super Stuff boluses of varying thicknesses (up to 20 mm) were compared with commercial boluses. Second, long-term stability was assessed over 65 days for dose delivery, thickness (via CT-based measurements), and CT number. Finally, in a clinical case of Ewing’s sarcoma, setup reproducibility and conformity were assessed using Radixact’s kVCT imaging. Delivered dose distributions were compared with the planned distribution using dose–volume histogram parameters and gamma analysis.

**Results:**

Surface dose measurements demonstrated that the maximum variation in surface dose among Super Stuff boluses with thicknesses ranging from 10.7 to 19.8 mm was within 4%. Over 65 days, the Super Stuff bolus showed good long-term stability. Changes in thickness were limited to a maximum of 1.6 mm, and fluctuations in CT number remained stable at 17.9 ± 1.2 Hounsfield units. In the clinical setting, kVCT imaging provided clear visualization of the Super Stuff bolus, and setup reproducibility was maintained throughout the treatment course. Air gaps were also minimized. Furthermore, gamma analysis (3%/2 mm) confirmed high dosimetric reproducibility throughout the treatment course, with passing rates exceeding 96% between the first and subsequent treatment fractions.

**Conclusions:**

The Super Stuff bolus provides notable clinical advantages for treating superficial tumors using Radixact with the kVCT system: consistent surface dose buildup, easy fabrication, and robust long-term physical and dosimetric stability. The seamless integration with the kVCT system enhances setup reproducibility, contributing to reliable and accurate dose delivery throughout the treatment course.

## 1 Introduction

Radixact is the latest TomoTherapy integrated system that combines imaging and treatment delivery. It was specifically designed to optimally implement intensity-modulated radiation therapy (IMRT) in clinical settings (1). IMRT using Radixact is currently applied in various clinical cases (2–5). Specifically, Radixact is a radiation therapy platform that exclusively uses photon beams. The Radixact delivery system features a compact 6 MV linear accelerator mounted on a ring gantry.

High-energy photon beams exhibit skin-sparing properties near the surface inside a patient (6–8). This occurs due to a dose build-up effect of megavoltage photon beams. While beneficial in many cases, this property poses challenges when treating superficial tumors located near the skin surface. For example, the skin surface receives approximately 70% of the prescribed dose in post-mastectomy irradiation (9).

Boluses are used as build-up materials to compensate for the reduced dose to the superficial areas (10–14). The boluses increase the skin surface dose and improve dose uniformity during the treatment of superficial tumors (15, 16). The effectiveness of boluses has also been studied in radiation therapy for superficial tumors treated with TomoTherapy (17, 18).

However, achieving full contact between commercially available flat boluses and an irregular patient skin surface is challenging. This may result in an air gap between the bolus and skin. Previous studies have reported that an undesirable air gap under the bolus reduces both dose coverage and homogeneity in the tumor volume (19–21). Although the impact on the dose depends on treatment energy, field size, bolus thickness, and air gap size, a 1-cm air gap reduces the surface dose by > 10% compared to the surface dose without an air gap (19, 22, 23).

One approach to addressing this issue is the development of customized boluses using three-dimensional (3D) printing technology. Previous studies (24–28) have demonstrated that patient-specific boluses can minimize air gaps, leading to decreased dose uncertainty and heterogeneity. However, several concerns remain regarding their clinical application. 3D-printed boluses are typically rigid because they are made of plastic materials (27, 29). This rigidity leads to non-conformity to the body surface in the inframammary fold (27) and non-adaptation to the daily variation of the body surface (22, 29, 30). Park et al. (27) highlighted that a plastic bolus may not conform to the body surface if the patient breathes during treatment. Furthermore, patients with sensitive skin or open wounds may experience discomfort due to the rigidity of the plastic bolus (27, 29, 31).

Recently, customized boluses made from softer, highly conformable silicone materials have been developed to address the limitations of plastic boluses (22, 30, 31). Compared with plastic boluses, these silicone-based custom boluses offer superior conformity to the skin and reduce dose uncertainty by minimizing air gaps (22, 30, 32). However, the time and effort required to fabricate the customized bolus with soft materials can be problematic in clinical application (22, 30–32). Patients may face treatment delays if the bolus needs to be remade due to reasons such as body shape changes or breakage during the treatment course.

Given the challenges of both rigid and soft customized boluses, we focused on the Super Stuff bolus that reduces fabrication time and provides flexibility. A prior work (33) has reported that this bolus provides a bolus effect similar to conventional paraffin wax boluses, with flexibility and ease of fabrication. However, under conventional, non-image-guided setup procedures, its flexible properties raise concerns regarding the accuracy and reproducibility of each setup, including the bolus thickness. We hypothesized that setup reproducibility could be adequately ensured using the kilovoltage computed tomography (kVCT) system integrated with Radixact, which provides excellent high-quality image visibility. Although the Super Stuff bolus has been used in conventional radiation therapy, to the best of our knowledge, no previous studies have evaluated the clinical utility or reproducibility of the Super Stuff bolus in combination with this integrated kVCT system.

Therefore, this study aimed to assess the applicability of the Super Stuff bolus during the treatment of superficial tumors using Radixact with the kVCT system. Specifically, the variation of surface dose with bolus thickness was first evaluated through phantom experiments. The stability of the Super Stuff bolus over time was subsequently examined in terms of surface dose, shape, and CT number. Finally, we assessed the visibility and adherence of the Super Stuff bolus to the body surface using the kVCT system in an actual clinical case. The variation from the planned dose was quantified by comparing the planned and delivered dose distributions.

## 2 Materials and methods

### 2.1 Measurement of the surface dose for various bolus thicknesses

**Figure 1:**
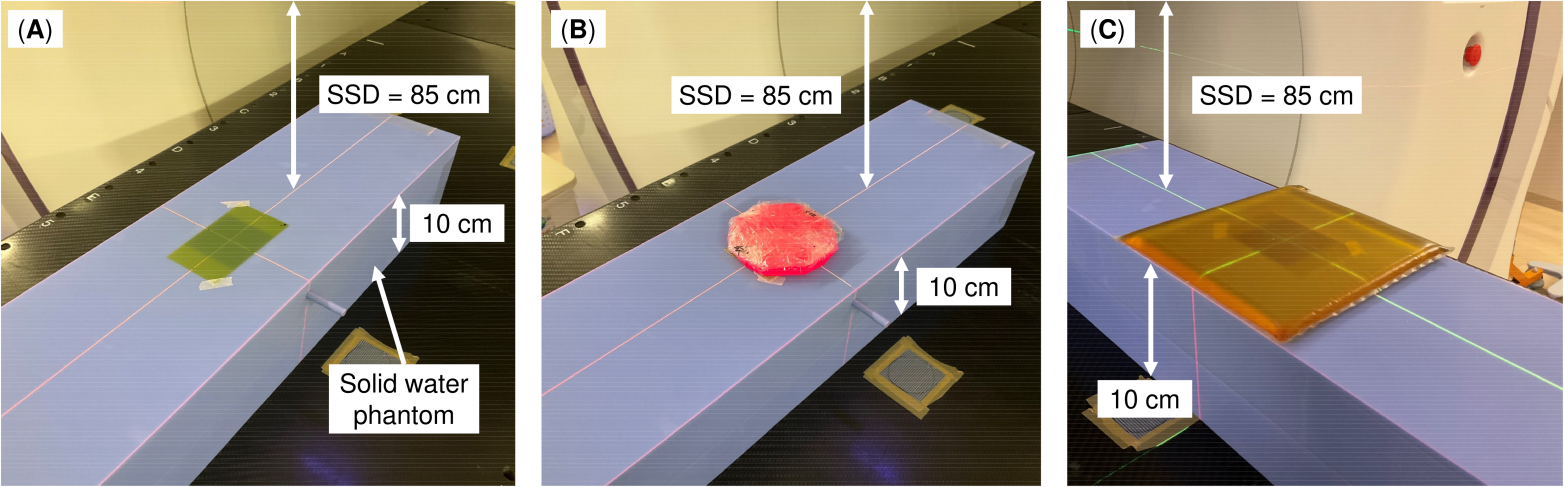
Measurement geometry. **(A)** No bolus, **(B)** Super Stuff bolus, and **(C)** commercial bolus. The source–surface distance (SSD) represents the distance from the source to the phantom surface, not the distance from the source to the bolus surface.

The Super Stuff bolus was fabricated following the manufacturer’s instructions (34). Six distinct boluses, each with thicknesses of up to 20 mm, were fabricated, wrapped in plastic, and molded. The wrapping thickness is approximately 10 μm, which is equivalent to approximately 0.01 mm of water-equivalent thickness. Therefore, its impact on the measured dose for 6 MV photon beams is considered negligible. One bolus was fabricated for each thickness, and these thicknesses were randomly set to be approximately 3 mm apart. Measurements were obtained using a Radixact-X9 machine (Accuray, Sunnyvale, CA, USA) equipped with a 6 MV flattening-filter-free photon beam. Irradiation conditions were set by constructing a machine quality assurance procedure on the treatment delivery console (TDC version 3.0.3, Accuray). The gantry angle was set to 0° (“static”), and the field size was 5 × 5 cm² (i.e., field width = 5 cm). Additionally, the delivery time was 11 s, and the couch remained fixed. **Figure 1** shows the measurement geometry. A radiochromic film (GAF-CHROMIC EBT3; Ashland, KY, USA) was placed on the surface of a solid water HE phantom (55 × 15 × 5 cm^3^; Gammex, Middleton, WI, USA). Subsequently, the Super Stuff bolus was placed to completely cover the radiochromic film. The distance from the source to the phantom surface was 85 cm. Measurements were also obtained under a condition without a bolus (hereafter, no-bolus condition) to confirm the build-up effect of the Super Stuff bolus. To compare the build-up effect of commercial boluses, additional measurements were obtained by placing 5- and 10-mm thick commercial boluses (Bolus 31051 and Bolus 31101, respectively; CQ Medical, Avondale, PA, USA) instead of the Super Stuff bolus. Each measurement was performed in triplicate. Films were scanned using a VIDAR DosimetryPRO Advantage (Red) system (VIDAR Systems Corporation, Herndon, VA, USA) and analyzed using the RIT image analysis software (version 6.10, Radiological Imaging Technology, Colorado Springs, CO, USA). The time interval between irradiation and film scanning was consistent across all measurements. Film densities were converted to doses using a density-to-dose conversion table created with the same film lot used in the measurements. This table was established by irradiating a series of films with known doses up to 500 cGy under 5 × 5 cm² field size conditions at 5 cm depth (source-surface distance = 85 cm), and the resultant calibration curve was derived using at least 13 data points in accordance with the Task Group 69 report by the American Association of Physicists in Medicine (35). Dose values were averaged within a 3 × 3 cm^2^ region of interest (ROI) defined within the irradiation field.

### 2.2 Evaluation of the long-term stability of the super stuff bolus

Repeated measurements were performed to evaluate the long-term stability of the Super Stuff bolus over time in terms of surface dose, shape, and CT number. For each of these items, the evaluation was performed for 65 days from the date of the bolus fabrication. This period is sufficient to evaluate stability during treatment courses. The boluses were maintained under uniform conditions (temperature: 20°C, humidity: 35%) and wrapped in plastic. However, the boluses were wrapped in plastic but were not subjected to strict environmental control during irradiation and scanning, considering the short duration required for the procedures.

#### 2.2.1 Evaluation of the surface dose

The measurements were repeated using the same procedure as described in Section 2.1. Specifically, the phantom was placed in the same position on the couch, and the couch height was set to the same height each time to reduce uncertainty in the measurement procedure. The procedure from scanning to analyzing the irradiated film was consistent across all measurements. Measurements were obtained on days 1, 2, 9, 16, 26, 36, and 65 after bolus fabrication (seven times in total). Measurements without a bolus were also performed to evaluate the stability of the machine’s output and reproducibility of the measurement procedure.

#### 2.2.2 Evaluation of the bolus thickness

The bolus thickness was measured as part of the shape evaluation. The bolus was scanned via CT each time, and the images were analyzed to determine its thickness. First, the images were transferred to the RayStation treatment planning system (version 10.0.1, RaySearch, Stockholm, Sweden). Subsequently, the bolus was delineated using the body contouring function in RayStation, with application of a default CT number threshold of −250 Hounsfield units (HU) to maintain consistency with clinical practice. A 1 × 1 cm² ROI was defined at the center of the bolus in the coronal plane, with the vertical direction (i.e., thickness direction) expanded to overlap with the bolus contour. Finally, the bolus thickness was derived by measuring the volume of the ROI created through the procedure described previously. We also assessed the validity of this method by deriving the thickness of commercial boluses using the same approach. Measurements were obtained on days 0, 1, 2, 5–9, 12–16, 20–22, 26, 28, 30, 36, 50, 57, and 65 after bolus creation (23 times in total). Because the measurement procedure was standardized and did not introduce measurement variability, only one measurement was performed for each observation.

#### 2.2.3 Evaluation of the CT number

CT numbers of the bolus were measured using CT images acquired for bolus thickness evaluation. Additionally, the ROI size for bolus thickness evaluation was expanded to 3 × 3 cm², and average CT numbers within this ROI were measured using RayStation. Measurements were obtained on the same days as those for the bolus thickness evaluation. As with the bolus thickness evaluation, only one measurement was performed for each observation because of the standardized measurement procedure.

### 2.3 Evaluation of super stuff boluses in clinical practice

Radixact, combined with the Super Stuff bolus, was used to irradiate metastatic inguinal lymph node lesions of Ewing’s sarcoma that extended to the skin surface. The bolus was wrapped and molded sufficiently to fully cover the lesion area, ensuring a uniform thickness of 15 mm. The bolus was stored during the treatment course as described in Section 2.2. The 15-mm thickness was determined based on the findings of Section 2.1. Written informed consent was obtained from the patient, all data were anonymized, and the Institutional Ethics Committee of the Anjo Kosei Hospital approved this study (ID: R24-036).

#### 2.3.1 Reproducibility of setups using the kVCT system

During each treatment session, the bolus was positioned according to the markers defined on the skin surface during the simulation. It was firmly pressed against the skin surface to reduce the air gap between them. The reproducibility of bolus setup (position and thickness) and air gap was evaluated using CT images acquired by the kVCT system immediately before irradiation. At treatment planning, a 5-mm enlarged or reduced contour of the bolus (only the surface not in contact with the body surface) was created in addition to the bolus contour and used as the basis for evaluation. This approach enabled quantitative evaluation. The enlargement or reduction of 5 mm was determined based on the findings of Section 2.1. Furthermore, the inter- and intra-observer variabilities were controlled by displaying the 5-mm enlarged or reduced contours for each treatment session and confirming that the placed bolus fell within these contours across all cross-sections of the CT images where the bolus was present. The absence of any large air voids was confirmed on CT images by visual assessment.

#### 2.3.2 Comparison of the dose distribution between the treatment plan and each treatment session

Treatment planning was conducted using RayStation, which has been commissioned for use with Radixact. The helical tomotherapy plan was optimized with a field width of 2.5 cm and a pitch of 0.303, using a dose calculation grid size of 2.0 mm. The modulation factor was 2.1. These planning parameters were selected to balance treatment time and dose conformity. For planning target volume (PTV), the prescribed dose was 5,000 cGy in 25 fractions. The dose was normalized using 95% of the PTV with 5,000 cGy. The PTV was defined as a 10-mm isotropic expansion of the gross tumor volume (GTV) in accordance with our institutional protocol. The CT images acquired by the kVCT system were transferred to RayStation, where the dose distribution for each treatment fraction was calculated. A conversion table created with an electron density phantom (Model 062 MA, CIRS, Norfolk, VA, USA) containing 12 density plugs of known composition was used to convert CT numbers to electron density. The kVCT images used to create this conversion table were acquired using the same imaging protocol used in clinical practice. The validity of this conversion table was confirmed by comparing it with previously published data (36). Furthermore, the delivered dose distributions from the first, tenth, and twenty-fifth treatment fractions were compared with the planned dose distribution to evaluate the dose distribution reproducibility. Dose–volume histogram parameters for GTV, along with gamma analysis performed using the RIT image analysis software, were used to compare the planned and delivered dose distributions.

### 2.4 Statistical analysis

All statistical analyses were performed to evaluate the performance and stability of the Super Stuff bolus. Data are presented as mean ± standard deviation, unless otherwise stated.

- Surface dose comparisons: Two-sided Student’s *t*-tests were used to compare the surface dose measurements between the Super Stuff bolus at each thickness and the commercial boluses.
- Long-term surface dose stability: Surface dose stability was assessed by comparing the variance of repeated measurements over 65 days. *F*-tests were used to compare the variance of measurements with the Super Stuff bolus against the variance of measurements without a bolus (representing the baseline stability of the measurement system).
- Bolus shape stability: The homogeneity of variances in repeated thickness measurements across the different bolus groups was examined using Bartlett’s test to evaluate the long-term stability of the bolus shape.

All statistical tests were two-sided, with statistical significance set at p < 0.05. Microsoft Excel (Microsoft Corporation, Redmond, WA, USA) and R software (version 4.3.2; R Foundation for Statistical Computing, Vienna, Austria) with the stats package were used to perform all analyses.

## 3 Results

### 3.1 Measurement of the surface dose for various bolus treatments

**Figure 2:**
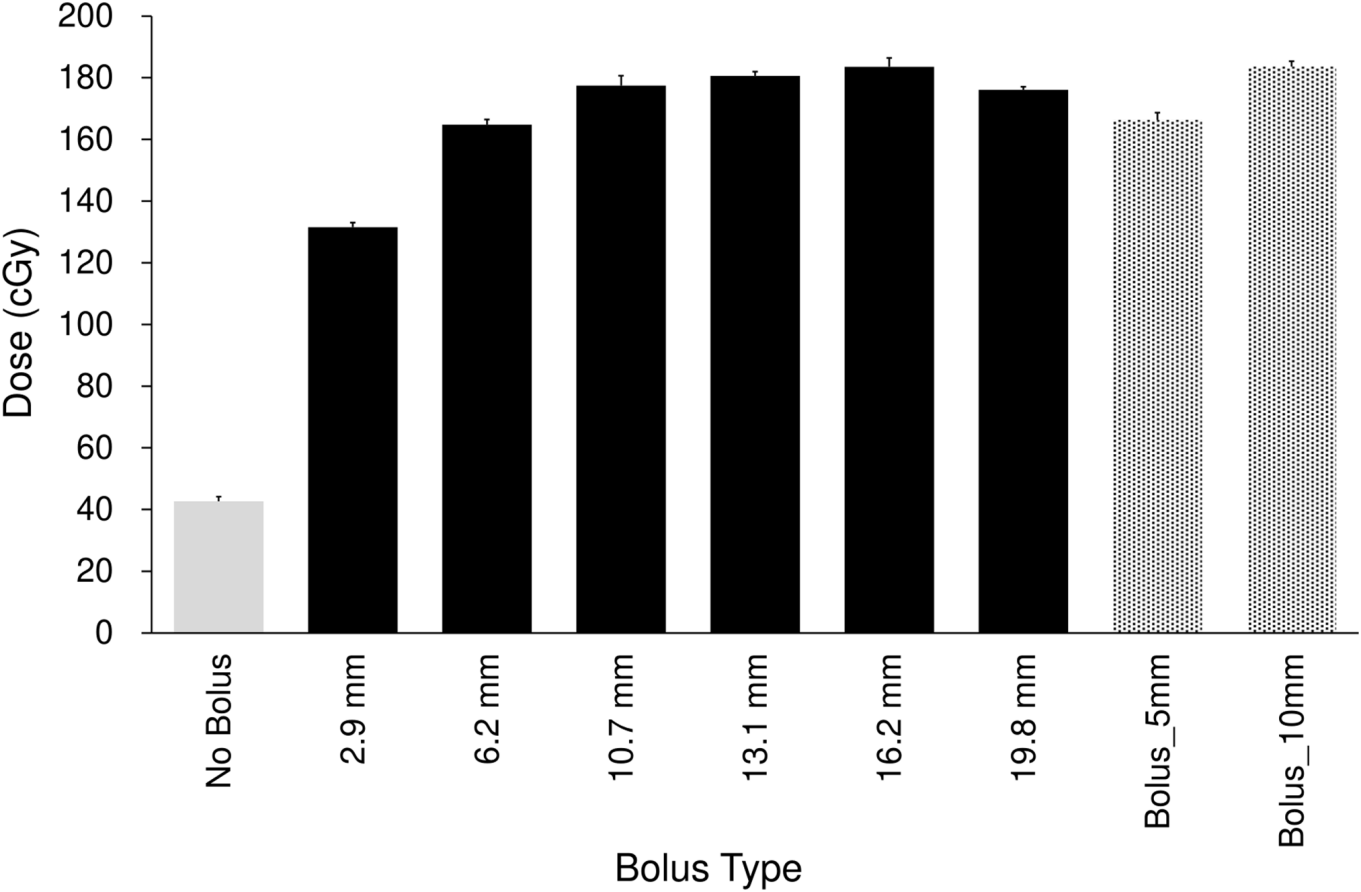
Comparison of surface doses for the no-bolus condition, six Super Stuff boluses (thicknesses: 2.9, 6.2, 10.7, 13.1, 16.2, and 19.8 mm), and two commercial boluses (5 and 10 mm). Error bars represent one standard deviation of each measurement.

Each Super Stuff bolus could be fabricated in approximately 30 min. **Figure 2** and **Table 1** show the surface dose measurement results for the no-bolus condition, six Super Stuff boluses of varying thicknesses, and two commercial boluses. The measured value for the no-bolus condition was 42.67 ± 1.54 cGy. For the six Super Stuff boluses with thicknesses of 2.9, 6.2, 10.7, 13.1, 16.2, and 19.8 mm, the measured values were 131.53 ± 1.54, 164.82 ± 1.67, 177.49 ± 3.17, 180.60 ± 1.40, 183.52 ± 2.88, and 176.09 ± 0.97 cGy, respectively. The surface dose increased with bolus thickness up to 16.2 mm and remained nearly constant between 10.7 and 19.8 mm. Similarly, the measured values for the commercial boluses with thicknesses of 5 and 10 mm were 166.34 ± 2.36 and 183.61 ± 1.73 cGy, respectively. The 16.2-mm-thick Super Stuff bolus demonstrated a 4.3-fold build-up effect compared to the measured value of the no-bolus condition, which is nearly equivalent to that of the 10-mm-thick commercial bolus. No significant difference was found between these two boluses (p = 0.973). The maximum difference in surface dose across the Super Stuff boluses with thicknesses measured from 10.7 to 19.8 mm was 7.43 cGy. This value corresponded to a 4% difference based on the surface dose of the 16.2-mm-thick Super Stuff bolus.

**Table 1:** Comparison of surface doses for the no-bolus condition, six Super Stuff boluses (thicknesses: 2.9, 6.2, 10.7, 13.1, 16.2, and 19.8 mm), and two commercial boluses (5 and 10 mm).

Bolus type	Thickness (mm)	Surface dose (cGy)
No Bolus	N/A	42.67 ± 1.54
Super Stuff Bolus	2.9	131.53 ± 1.54
	6.2	164.82 ± 1.67
	10.7	177.49 ± 3.17
	13.1	180.60 ± 1.40
	16.2	183.52 ± 2.88
	19.8	176.09 ± 0.97
Commercial Bolus	5	166.34 ± 2.36
	10	183.61 ± 1.73

### 3.2 Evaluation of the long-term stability of the super stuff bolus

#### 3.2.1 Evaluation of the surface dose

**Figure 3:**
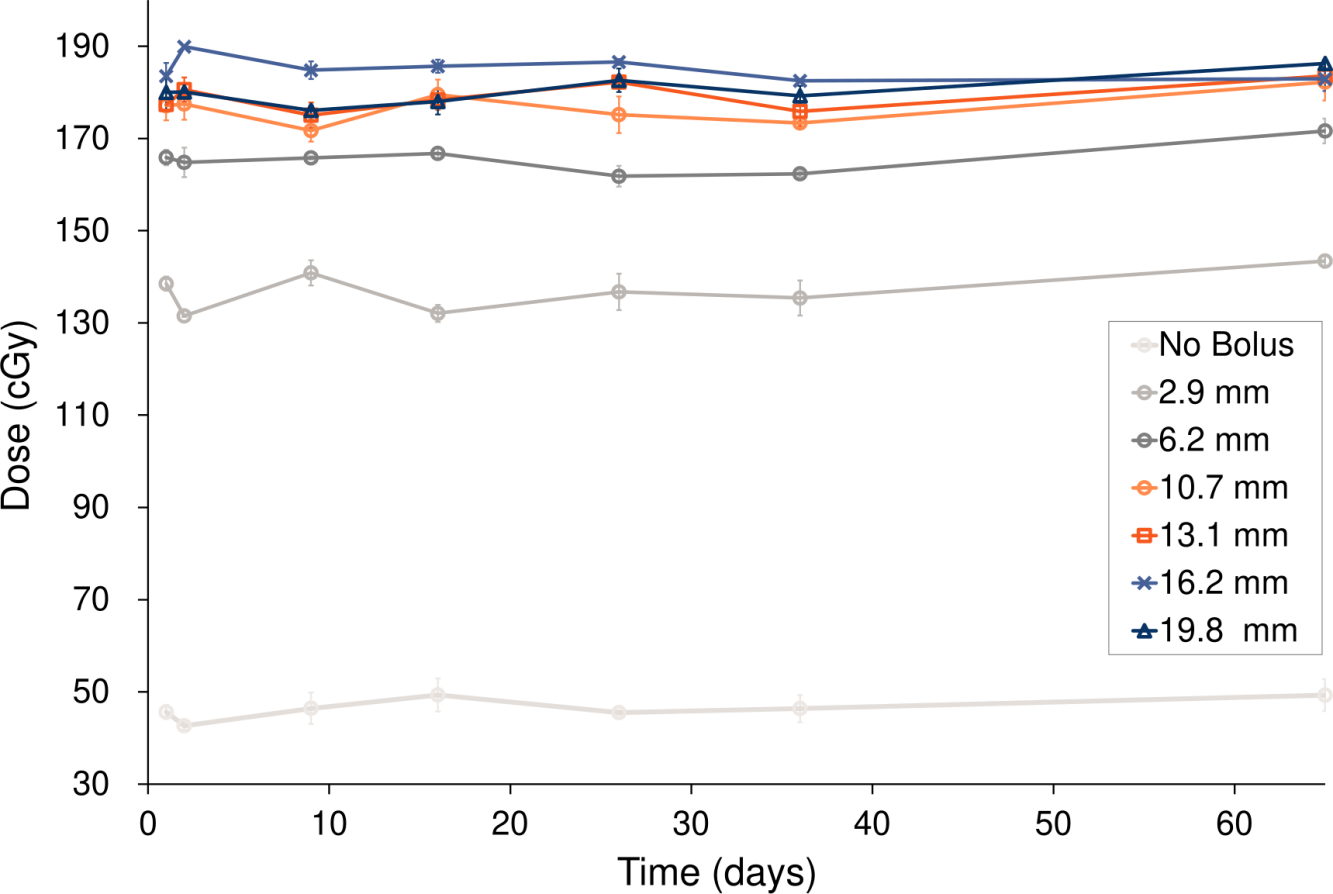
Surface dose variations over 65 days for the six Super Stuff boluses of distinct thicknesses. Error bars represent one standard deviation of each measurement.

**Figure 3** and **Table 2** show the changes in surface dose over time for the six different Super Stuff boluses, measured seven times over 65 days. The measured value for the no-bolus condition was 46.47 ± 2.14 cGy. These values represent the mean and standard deviation (SD), respectively, of the seven daily measurements performed over 65 days. Each daily measurement is the average of three repeated readings. The average SD of these intra-day repeated readings was ± 2.05 cGy. For the six Super Stuff boluses with thicknesses of 2.9, 6.2, 10.7, 13.1, 16.2, and 19.8 mm, the measured values were 136.93 ± 4.05, 165.57 ± 3.01, 176.64 ± 3.32, 179.00 ± 2.99, 185.14 ± 2.39, and 180.33 ± 3.04 cGy, respectively. The maximum dose difference observed over 65 days for the no-bolus condition was 6.65 cGy. For the six Super Stuff boluses with thicknesses of 2.9, 6.2, 10.7, 13.1, 16.2, and 19.8 mm, the dose differences were 11.87, 9.82, 10.50, 8.52, 7.42, and 10.16 cGy, respectively. The long-term variability of surface dose measurements did not significantly differ between the Super Stuff boluses and the no-bolus condition. Specifically, the p-values from the variance comparison were 0.173, 0.251, 0.432, and 0.223 for the 10.7-, 13.1-, 16.2-, and 19.8-mm boluses, respectively.

**Table 2:** Surface dose variations over 65 days for the six Super Stuff boluses of distinct thicknesses.

Time (days)	Surface dose (cGy)						
	No bolus	Super stuff bolus					
		2.9 mm	6.2 mm	10.7 mm	13.1 mm	16.2 mm	19.8 mm
1	45.64	138.51	165.86	177.11	177.35	183.52	179.99
2	42.67	131.53	164.82	177.49	180.60	189.93	180.09
9	46.42	140.85	165.79	171.73	175.04	184.81	176.09
16	49.32	132.08	166.76	179.47	178.35	185.67	178.03
26	45.51	136.74	161.18	175.14	182.24	186.59	182.61
36	46.41	135.41	162.33	173.34	175.87	182.51	179.24
65	49.30	143.40	171.63	182.22	183.56	182.91	186.25
Mean	46.47	136.93	165.57	176.64	179.00	185.14	180.33
SD	2.14	4.05	3.01	3.32	2.99	2.39	3.04

All values measured on days 1–65 represent the averages of three measurements performed at each observation day. The "Mean" and "SD" rows indicate the mean and standard deviation of these seven observation days, respectively.

#### 3.2.2 Evaluation of the bolus thickness

**Figure 4:**
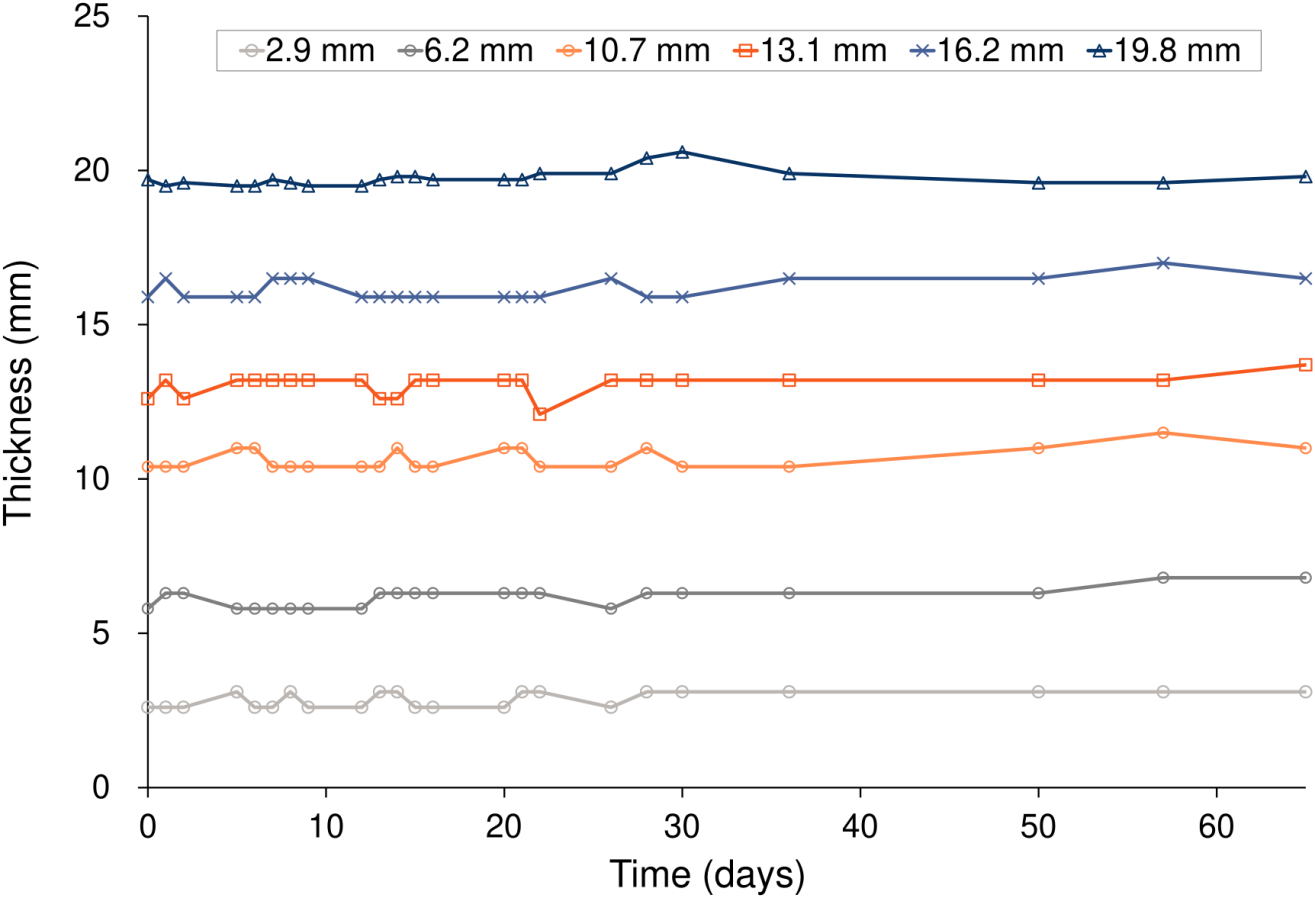
Thickness variations over 65 days for the six Super Stuff boluses of different initial thicknesses.

**Figure 4** and **Table 3** show the changes in thickness for six different Super Stuff boluses, measured 23 times over 65 days. The maximum changes in thickness were 0.5, 1.0, 1.1, 1.6, 1.1, and 1.1 mm at thicknesses of 2.9, 6.2, 10.7, 13.1, 16.2, and 19.8 mm, respectively. No significant difference was found in the variances of the thickness measurements across the bolus groups (p = 0.652). For the 5- and 10-mm commercial boluses, the thicknesses measured were 5.0 and 10.2 mm, respectively.

**Table 3:** Thickness variations over 65 days for the six Super Stuff boluses of different initial thicknesses.

Time (days)	Thickness (mm)					
	Super stuff bolus					
	2.9 mm	6.2 mm	10.7 mm	13.1 mm	16.2 mm	19.8 mm
0	2.6	5.8	10.4	12.6	15.9	19.7
1	2.6	6.3	10.4	13.2	16.5	19.5
2	2.6	6.3	10.4	12.6	15.9	19.6
5	3.1	5.8	11.0	13.2	15.9	19.5
6	2.6	5.8	11.0	13.2	15.9	19.5
7	2.6	5.8	10.4	13.2	16.5	19.7
8	3.1	5.8	10.4	13.2	16.5	19.6
9	2.6	5.8	10.4	13.2	16.5	19.5
12	2.6	5.8	10.4	13.2	15.9	19.5
13	3.1	6.3	10.4	12.6	15.9	19.7
14	3.1	6.3	11.0	12.6	15.9	19.8
15	2.6	6.3	10.4	13.2	15.9	19.8
16	2.6	6.3	10.4	13.2	15.9	19.7
20	2.6	6.3	11.0	13.2	15.9	19.7
21	3.1	6.3	11.0	13.2	15.9	19.7
22	3.1	6.3	10.4	12.1	15.9	19.9
26	2.6	5.8	10.4	13.2	16.5	19.9
28	3.1	6.3	11.0	13.2	15.9	20.4
30	3.1	6.3	10.4	13.2	15.9	20.6
36	3.1	6.3	10.4	13.2	16.5	19.9
50	3.1	6.3	11.0	13.2	16.5	19.6
57	3.1	6.8	11.5	13.2	167.0	19.6
65	3.1	6.8	11.0	13.7	16.5	19.8
Mean	2.9	6.2	10.7	13.1	16.2	19.8
SD	0.2	0.3	0.3	0.3	0.3	0.3

The "Mean" and "SD" rows indicate the mean and standard deviation of these 23 observation days, respectively.

#### 3.2.3 Evaluation of the CT number

**Figure 5:**
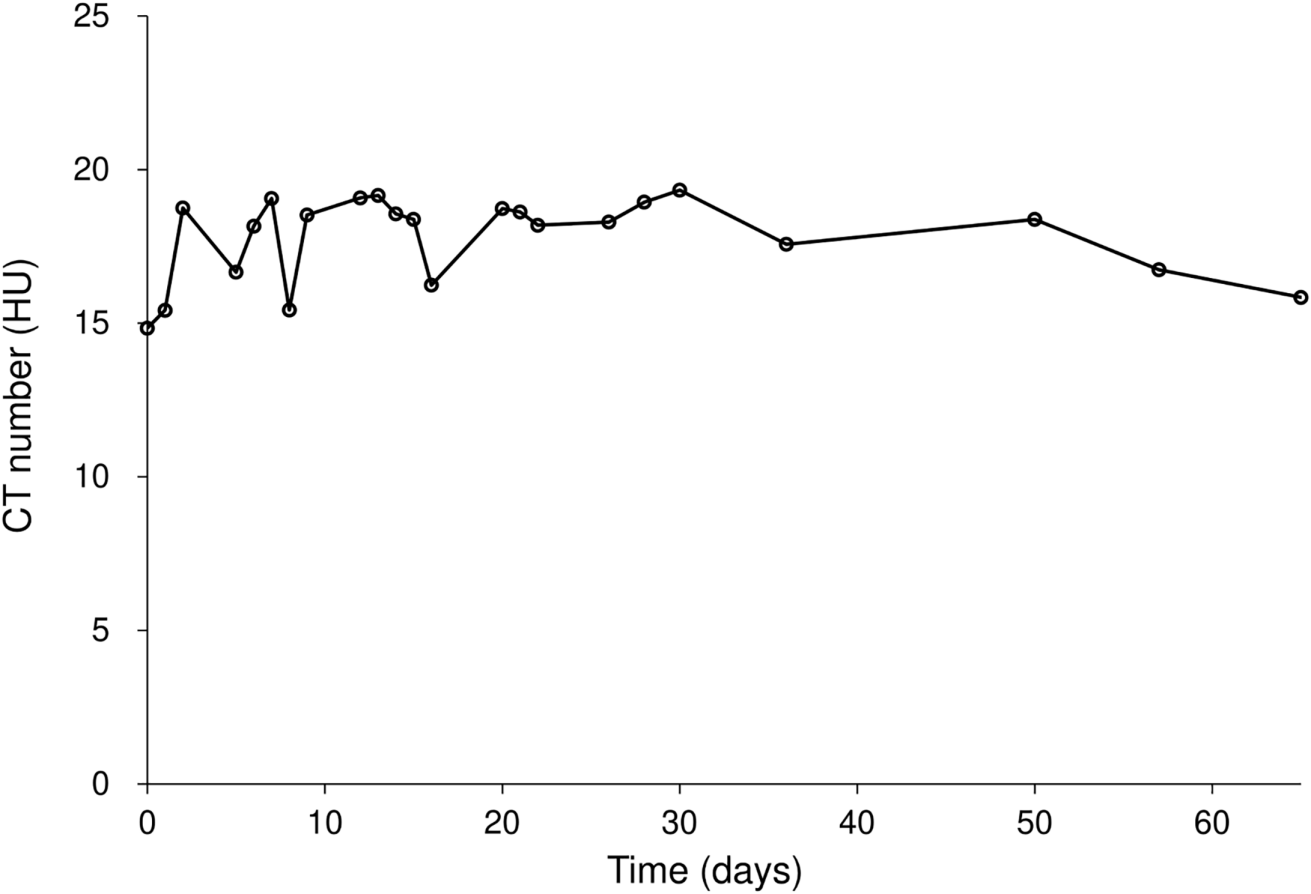
CT number variation for the Super Stuff bolus over 65 days, with the 16.2-mm thickness presented as representative. CT: computed tomography.

**Figure 5** and **Table 4** present the changes in the CT number of the Super Stuff bolus measured 23 times over 65 days. Due to minimal differences in CT numbers across various thicknesses, the results for the 16.2-mm thickness are presented as representative. The average CT value was 17.9 ± 1.2 HU, with a maximum CT number difference of 3.9 HU over 65 days.

**Table 4:** CT number variation for the Super Stuff bolus over 65 days, with the 16.2-mm thickness presented as representative.

Time (days)	CT number (HU)
0	14.84
1	15.42
2	18.75
5	16.66
6	18.16
7	19.06
8	15.43
9	18.52
12	19.08
13	19.16
14	18.56
15	18.38
16	16.24
20	18.73
21	18.62
22	18.19
26	18.29
28	18.94
30	19.33
36	17.57
50	18.38
57	16.74
65	15.84
Mean	17.9
SD	1.2

The "Mean" and "SD" rows indicate the mean and standard deviation of these 23 observation days, respectively.

### 3.3 Evaluation of super stuff boluses in clinical practice

#### 3.3.1 Reproducibility of setups Using the kVCT system

**Figure 6:**
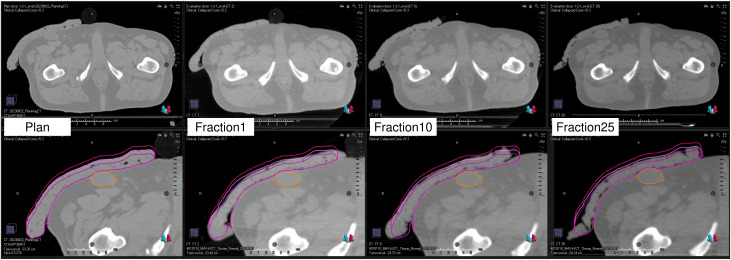
Computed tomography image used for treatment planning and kilovoltage computed tomography images acquired immediately before irradiation at the first, tenth, and twenty-fifth treatment fractions. The lower panels display the bolus contour, ± 5 mm modified contours (external surface only), and the gross tumor volume contour.

**Figure 6** illustrates the CT image used for treatment planning and the CT images acquired by the kVCT system immediately before irradiation at the first, tenth, and twenty-fifth treatment fractions. The images acquired by the kVCT system showed excellent visibility of the Super Stuff bolus, confirming that it was set up reproducibly, with thickness variations from the time of treatment planning maintained within 5 mm. The bolus was firmly pressed against the skin surface, minimizing the air gap between them.

#### 3.3.2 Comparison of the dose distribution between the treatment plan and each treatment session

**Figure 7:**
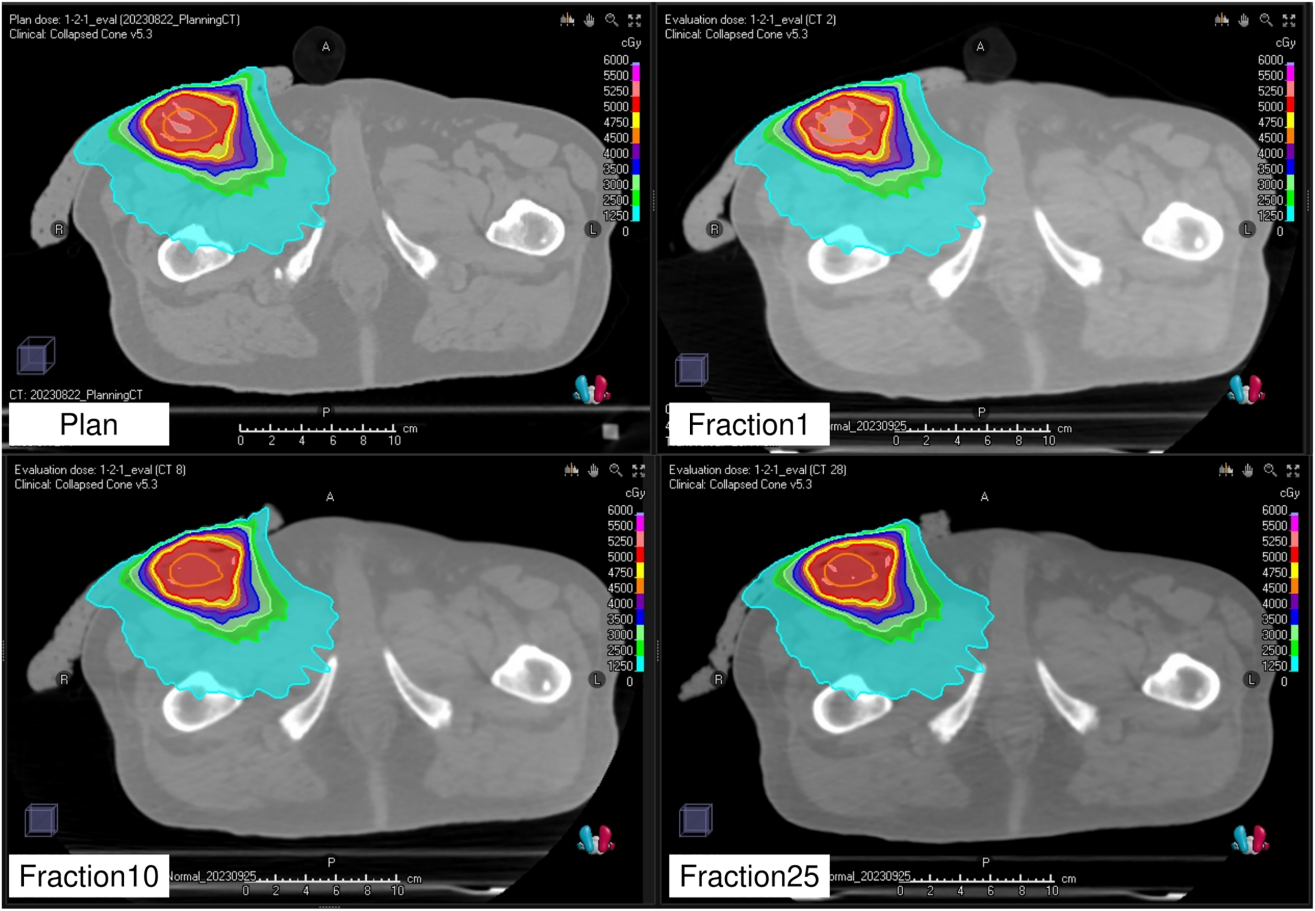
Planned dose distribution and recalculated dose distributions at the first, tenth, and twenty-fifth fractions.

**Figure 8:**
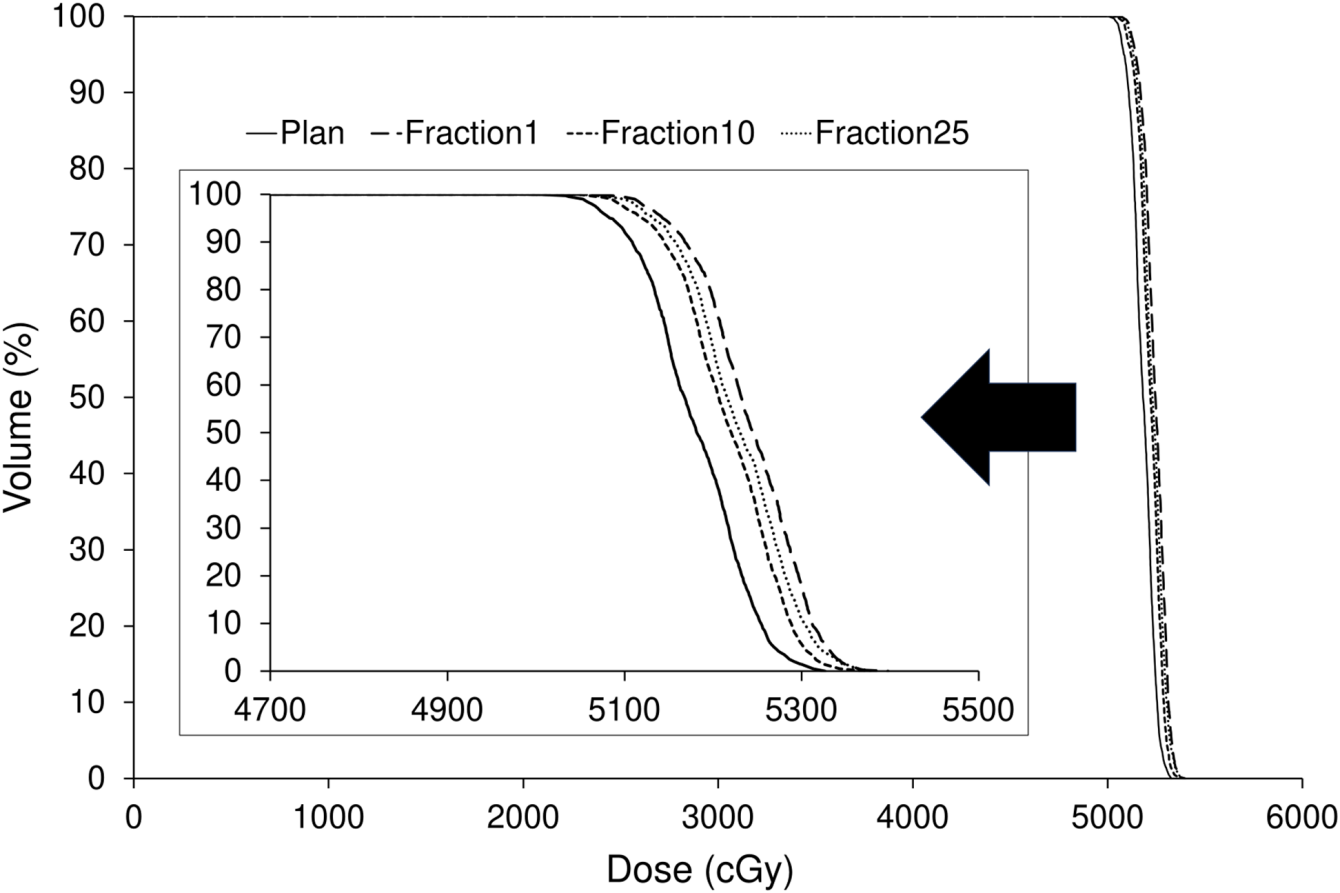
Comparison of dose–volume histograms for the gross tumor volume between the treatment plan and the first, tenth, and twenty-fifth treatment fractions.

**Figure 7** illustrates the dose distributions generated in the treatment plan, along with the recalculated dose distributions at the first, tenth, and twenty-fifth treatment fractions. **Figure 8** shows the differences in the dose–volume histogram for GTV. The difference in GTV mean dose compared to the treatment plan was 61, 36, and 48 cGy for the first, tenth, and twenty-fifth treatment fractions, respectively. These differences corresponded to 1.22%, 0.72%, and 0.96% of the prescribed dose, respectively. The gamma passing rates using the 3%/2 mm criterion were 93.63% for comparing the dose distribution between the treatment plan and the first treatment fraction, 96.26% between the first and tenth treatment fractions, and 98.19% between the first and twenty-fifth treatment fractions.

## 4 Discussion

We evaluated the effectiveness of Super Stuff boluses in treating superficial tumors using Radixact. The results revealed that the surface dose variation was reduced when using a bolus of 10–20 mm compared with that in cases involving an air gap. Build-up characteristics, shape (specifically thickness), and CT number remained stable over 65 days, indicating that it can be used across multiple treatment sessions. Furthermore, the use of the kVCT system contributed to improved reproducibility of the setup and minimized air gaps, thereby ensuring consistent delivery of the prescribed dose as intended in the treatment plan.

Super Stuff boluses were easily fabricated and showed sufficient build-up for 6 MV flattening-filter-free beams on the Radixact system (**Figure 2**). The effort required to fabricate a Super Stuff bolus is significantly lesser than that required to fabricate silicone-based custom boluses, which can take several days (30). Fabrication of a bolus using 3D printing technology is time-consuming and resource-intensive for patients and staff, as CT imaging is usually required before and after bolus fabrication. Super Stuff boluses allow for the quick initiation of radiation therapy without requiring additional labor. The density of the Super Stuff bolus is 1.02 g/cm³ (34), indicating that its build-up effect is comparable to that of commercial boluses. In this study, the surface dose of the Super Stuff bolus was slightly lower than that of a commercial bolus of the same thickness, likely due to tiny bubbles present in the Super Stuff bolus. However, in IMRT, small variations in surface dose can be managed through dose optimization.

The surface dose variation was approximately 4% when using Super Stuff boluses with thicknesses ranging from 10.7 to 19.8 mm (**Figure 2**). This minimal variation may be attributed to the tiny bubbles contained within the Super Stuff bolus, which decreases its effective density and might moderate the dose variation caused by differences in thickness. This finding indicates that even if the bolus is pressed against the skin surface at each treatment to minimize the air gap, the surface dose variation remains within 4% as long as the bolus thickness stays within this range. A dose variation of up to 4% is considered sufficiently small, especially when compared with the > 10% decrease in surface dose caused by an air gap (19, 22, 23). Additionally, measurements in this study were conducted using a single beam directed perpendicular to the bolus, representing the worst-case scenario in surface dosimetry (30). Therefore, the difference in surface dose due to bolus thickness is estimated as <4% for the commonly employed helical tomotherapy plan in IMRT using Radixact. Moreover, the surface dose due to the air gap decreases with a smaller field size, which is particularly significant in IMRT fields formed from many small field apertures (22, 23). Thus, the presence of an air gap has a significantly greater impact on surface dose when using the IMRT technique than when using the Super Stuff bolus with varying thickness (10–20 mm).

Concerns have been raised that Super Stuff boluses may crack and break during the treatment course, necessitating recreation due to deterioration (26). However, the results of the measurements over 65 days (**Figures 3**-**5**) effectively dispelled these concerns. In this study, changes in thickness and CT number were measured as physical properties in addition to changes in surface dose, which is the most important factor. This measurement approach enabled the evaluation of the stability of the Super Stuff bolus, independent of the uncertainties related to surface dosimetry. No observed changes in surface dose, thickness, or CT number were observed over time. To prevent the deterioration of Super Stuff boluses, wrapping them with plastic to avoid drying and storing them under consistent conditions are crucial. Although measuring surface doses with steep dose gradients proves challenging (37), the coefficient of variation for the measured surface doses in the no-bolus condition was 0.05 (**Figure 3**), suggesting reliable measurement results. Moreover, the coefficient of variations for the measured surface doses in Super Stuff boluses with thicknesses of 2.9, 6.2, 10.7, 13.1, 16.2, and 19.8 mm were 0.03, 0.02, 0.02, 0.01, and 0.02, respectively (**Figure 3**), indicating minimal changes in surface dose over time. The results of the F-tests comparing the variability of surface dose measurements without a bolus to those with Super Stuff boluses of varying thicknesses suggest that the surface dose remained stable across all tested bolus thicknesses. These findings suggest that applying Super Stuff boluses does not significantly affect the long-term consistency of surface dose measurements, thereby supporting their reliability in clinical settings. The thickness measurement method used in this study is considered appropriate, as the measurement error for a commercially available bolus was 0.2 mm. No changes were observed over time in the thickness of the Super Stuff bolus (**Figure 4**). Bartlett’s test showed non-significant results (p = 0.652), indicating consistent variability in bolus shape throughout the observation period, regardless of the bolus thickness. The CT number varied by up to 3.9 HU (**Figure 5**), a variation that exceeds the variability observed in the simultaneously measured commercial bolus (1.1 HU), possibly owing to bubbles in the Super Stuff bolus. However, based on the findings of previous studies (38, 39) that investigated how variations in CT number affect the accuracy of treatment planning dose calculations, a variation of 3.9 HU is unlikely to significantly impact the prescribed dose.

The kVCT system on Radixact has been reported to produce high-quality images (40), and even the Super Stuff bolus was clearly visualized (**Figure 6**). Similarly, the air gap between the bolus and skin surface was visualized, demonstrating the utility of the kVCT system in minimizing this gap. More flexible materials are better for fitting the bolus to the contours of the target site, which may change daily (29). The flexibility of the Super Stuff bolus also effectively minimized the air gap. Conversely, when the bolus is pressed against the skin surface to reduce the air gap, its thickness is expected to increase or decrease. However, in Radixact, the thickness of the bolus can be visualized during image registration by displaying virtual contours for both 10- and 20-mm bolus thicknesses, alongside the bolus contour from the treatment plan. This feature is a significant advantage in minimizing variations in surface dose. In addition, the reproducible setup of the Super Stuff bolus avoids covering healthy skin surfaces with the bolus, which can significantly reduce skin doses (22).

The dose distribution of the treatment plan was confirmed to be accurately reproduced by recalculation using the CT images acquired by the kVCT system at each treatment session (**Figure 7**). A comparison of the dose index between the original and recalculated plans at each treatment session showed a difference of approximately 1% (**Figure 8**). This difference was attributed to the fact that the plan at each treatment session was recalculated using CT images acquired by the kVCT system, aligning with the findings of a previous study (36). Consistent with prior observations, the gamma passing rate observed in the dose distribution comparison between the treatment plan and the first treatment fraction was lower than that observed between subsequent treatment fractions, supporting the implication that the Super Stuff bolus did not contribute to dose variation. Additionally, the variation in GTV mean dose between treatment fractions was below 0.5% (**Figure 8**), demonstrating that the prescribed dose was consistently delivered as planned throughout the treatment course. The higher gamma passing rates observed in comparisons between different treatment fractions further confirm the consistency of dose delivery. Although reports measuring surface doses in actual clinical patients exist (22), this study is the first to demonstrate the effectiveness of the Super Stuff bolus by evaluating the variation in dose delivered to the target throughout the treatment course.

This study focused primarily on evaluating the effectiveness of Super Stuff boluses. Although it did not specifically focus on their production reproducibility, boluses fabricated on different days exhibited minimal variation. Therefore, standardizing the methods of fabrication and storage is crucial to enhance the reproducibility of bolus characteristics for each production. A key factor influencing surface dose is the presence of bubbles in the bolus; however, large bubbles are visible on CT images used for treatment planning and can be removed by kneading the Super Stuff bolus again. The Radixact treatment planning system can achieve conformal and homogeneous dose distribution for superficial tumors, with or without a bolus, employing high tangential beamlet techniques (17). However, the accuracy of the Radixact dose calculation algorithm for superficial tumors has been reported as inaccurate (41, 42). Moreover, the dependence on tangential beamlets for irradiation makes the dose delivered to superficial tumors sensitive to patient positioning errors (17, 43). Notably, the use of Super Stuff boluses can overcome these issues.

This study has some limitations that should be acknowledged. First, our clinical evaluation did not include a direct comparison with commercially available boluses. However, a previous study investigating superficial pelvic tumors similar to those in our clinical case reported an air−gap volume of 169 cm³ beneath a commercial bolus in a patient undergoing treatment (30). Phantom experiments demonstrated that in regions with large air−gap volumes, the deviation between predicted and measured doses could reach up to 15.1% (30). Second, the clinical component of our study involved only a single patient. Therefore, future investigations should recruit a larger, preferably multicenter, cohort and incorporate direct, head-to-head comparisons with commercially available boluses to more robustly establish the clinical efficacy of the Super Stuff bolus.

In conclusion, we examined the effectiveness of Super Stuff boluses in treating superficial tumors using Radixact. The Super Stuff boluses exhibited minimal changes in physical properties throughout the treatment course. Additionally, the integrated kVCT system minimized surface dose variations by ensuring reproducible setup in terms of position and thickness while reducing the air gap. Therefore, Super Stuff boluses may facilitate consistent delivery of prescribed doses to superficial tumors, potentially enhancing treatment outcomes.

## Data Availability

The raw data supporting the conclusions of this article will be made available by the authors, without undue reservation.
